# O-Antigen Modulates Infection-Induced Pain States

**DOI:** 10.1371/journal.pone.0041273

**Published:** 2012-08-10

**Authors:** Charles N. Rudick, Mingchen Jiang, Ryan E. Yaggie, Vladimir I. Pavlov, Joseph Done, Charles J. Heckman, Christopher Whitfield, Anthony J. Schaeffer, David J. Klumpp

**Affiliations:** 1 Department of Urology, Feinberg School of Medicine, Northwestern University, Chicago, Illinois, United States of America; 2 Department of Physiology, Feinberg School of Medicine, Northwestern University, Chicago, Illinois, United States of America; 3 Department of Physical Medicine and Rehabilitation, Feinberg School of Medicine, Northwestern University, Chicago, Illinois, United States of America; 4 Department of Microbiology-Immunology, Feinberg School of Medicine, Northwestern University, Chicago, Illinois, United States of America; 5 Department of Molecular and Cellular Biology, University of Guelph, Guelph, Ontario, Canada; Kunming Institute of Zoology, Chinese Academy of Sciences, China

## Abstract

The molecular initiators of infection-associated pain are not understood. We recently found that uropathogenic *E. coli* (UPEC) elicited acute pelvic pain in murine urinary tract infection (UTI). UTI pain was due to *E. coli* lipopolysaccharide (LPS) and its receptor, TLR4, but pain was not correlated with inflammation. LPS is known to drive inflammation by interactions between the acylated lipid A component and TLR4, but the function of the O-antigen polysaccharide in host responses is unknown. Here, we examined the role of O-antigen in pain using cutaneous hypersensitivity (allodynia) to quantify pelvic pain behavior and using sacral spinal cord excitability to quantify central nervous system manifestations in murine UTI. A UPEC mutant defective for O-antigen biosynthesis induced chronic allodynia that persisted long after clearance of transient infections, but wild type UPEC evoked only acute pain. *E. coli* strains lacking O-antigen gene clusters had a chronic pain phenotype, and expressing cloned O-antigen gene clusters altered the pain phenotype in a predictable manner. Chronic allodynia was abrogated in TLR4-deficient mice, but inflammatory responses in wild type mice were similar among *E. coli* strains spanning a wide range of pain phenotypes, suggesting that O-antigen modulates pain independent of inflammation. Spinal cords of mice with chronic allodynia exhibited increased spontaneous firing and compromised short-term depression, consistent with centralized pain. Taken together, these findings suggest that O-antigen functions as a rheostat to modulate LPS-associated pain. These observations have implications for an infectious etiology of chronic pain and evolutionary modification of pathogens to alter host behaviors.

## Introduction

The mechanisms by which infections induce pain are not understood. Although inflammation may cause or contribute to pain [1], [2], few studies have examined infection-associated pain. UTI is the second-most common infectious disease, afflicting half of all women during their lifetime and costing billions of dollars in annual treatment costs in the U.S. [3], [4]. Most UTIs are caused by UPEC and result in pelvic pain and voiding dysfunction [5]. Interactions between UPEC and the urothelium that lines the bladder lumen result in local cytokine/chemokine production and accumulation of urinary IL-6 and IL-8 [6], [7]. IL-8 secretion, in turn, drives a robust innate immune response characterized by an influx of neutrophils that clear bacteria from the bladder. Yet it remains unclear how these events relate to pain.

An initial study of pain in murine UTI found that UPEC resulted in peripheral thermal sensitivity, measure of pain, that was abrogated in TLR4-deficient mice [8]. Since TLR4 mediates bladder responses to LPS, a major inflammatory mediator, these findings suggested UTI pain is due to inflammation. However, we recently examined pain during acute UTI by quantifying mechanical allodynia of the pelvic region, since visceral pain is manifested as cutaneous hypersensitivity [9]. Although UPEC induced TLR4-dependent acute allodynia, allodynia was not correlated with inflammation. Indeed, LPS purified from an asymptomatic bacteriuria (ASB) *E. coli* strain and from UPEC elicited similar inflammatory responses, but UPEC LPS produced allodynia whereas ASB LPS did not. These findings suggested that TLR4-dependent infection pain is a function of LPS structure independent of inflammation.

LPS is comprised of three major domains: the acylated lipid A component embedded in the outer membrane, a core oligosaccharide, and the O-antigen polysaccharide. The lipid A component is the chief mediator of inflammation through its interactions with TLR4 [10], [11], [12]. However, gram-negative bacteria derive significant diversity from differential forms of LPS, and *E. coli* generate much diversity at the level of O-antigen, where more than 180 serotypes have been identified. Although this diversity suggests functional importance for interactions between *E. coli* and hosts, the known functions of O-antigen are largely limited to enhancing bacterial robustness [13], [14], [15]. Since UPEC and ASB LPS elicit markedly different pain responses despite similar inflammation, we hypothesized that O-antigen modulates *E. coli* infection pain. Here, we demonstrate that O-antigen modulates TLR4-dependent pain phenotypes of *E. coli*, suggesting a novel target for pain control.

## Results

### Transient bacterial infection induces acute or chronic allodynia

**Figure 1:**
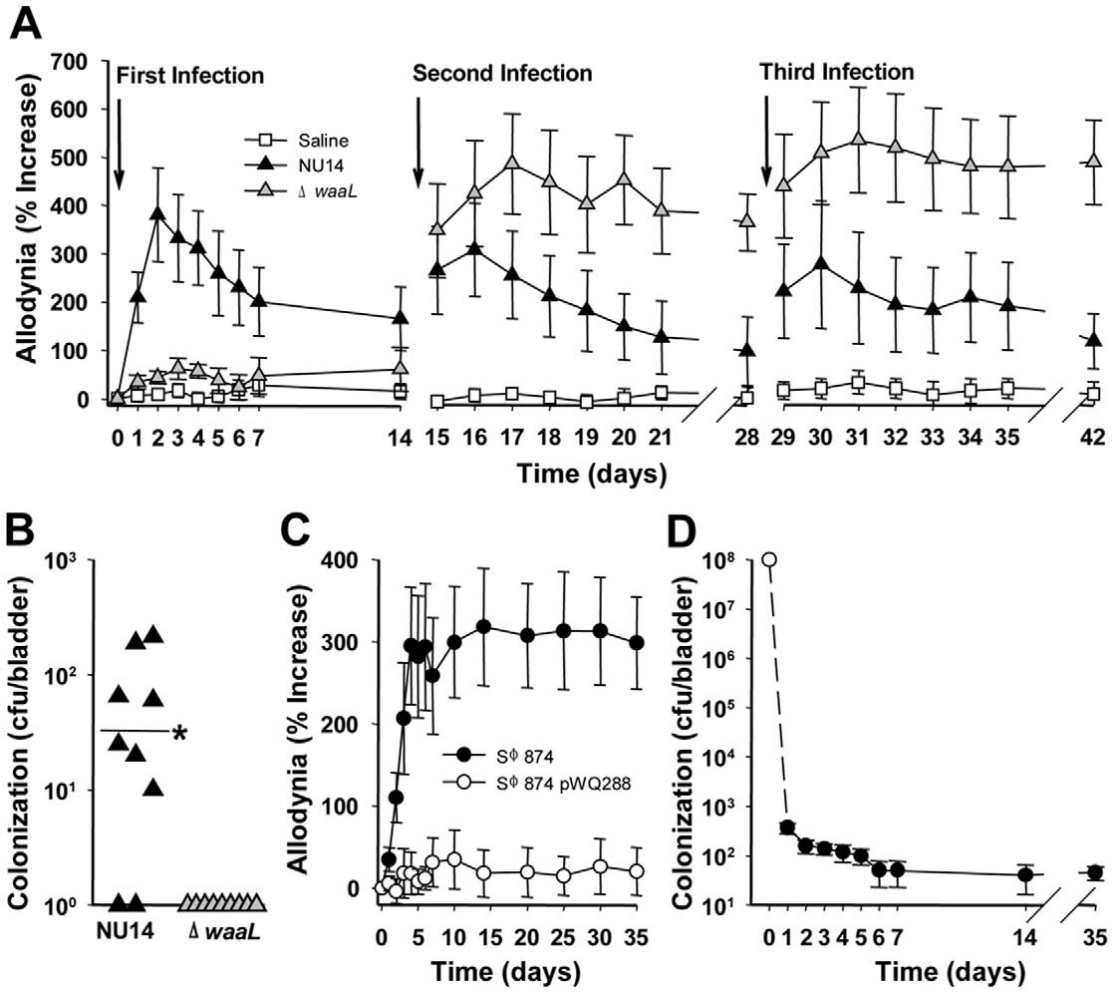
TLR4 mediates chronic pain. UTI was induced in female mice by instilling 10^8^ *E. coli* into the bladder, and tactile allodynia and bladder colonization were quantified. (**A**) Mice were instilled repeatedly with saline, NU14, or Δ*waaL* (n = 9). NU14 induced resolving acute pain (P<0.001 Days 2–5, P<0.01 Days 1 and 6, P<0.05 Days 7–10), but Δ*waaL* induced chronic pain following the second infection. (**B**) Bladders from mice in (A) had detectable NU14 colonization but not Δ*waaL* colonization (P<0.044). (**C**) SΦ874 pain (n = 10) was abrogated in mice infected with SΦ874 pWQ288 (n = 10). (**D**) SΦ874 is cleared rapidly from the bladder; open circle indicates inoculum (n = 5 Days 1–14, n = 10 Day 35).

We previously generated an O-antigen biosynthesis mutant in UPEC by deleting the gene encoding O-antigen ligase, *waaL*, from the archetypal cystitis isolate NU14 [16]. NU14Δ*waaL* is highly attenuated in murine UTI and represents a convenient strain to test the role of O-antigen in pain. Pain behavior was quantified using the classic approach of tactile allodynia in response to mechanical stimulation with calibrated von Frey filaments applied to the pelvic region [9]. Responses of jumping, abdominal retraction, or pelvic grooming were quantified relative to baseline, and mice that were infected with NU14 via transurethral catheter exhibited acute allodynia (Fig. 1A). Allodynia was greatest at 2 days post-infection and decayed over approximately two weeks, consistent with our previous observations [17], and serial NU14 infections also induced allodynia.

In contrast to wild type UPEC, serial infection with NU14Δ*waaL* induced chronic allodynia following a second infection and third infection (P<0.05 and P<0.01, respectively) despite evoking no response to an initial infection (Fig. 1A). Allodynia was specific to the pelvic region and not observed in the hind paw, and the absence of altered body mass suggested that overall health was not affected (Tables S1 and S2). Adaptive immune responses were next examined as potential mediators of chronic pain following serial infection. Rag1-deficient mice lacking T and B cell responses developed chronic pain in response to Δ*waaL*, and adoptive transfer of CD90+ splenocytes from mice with chronic allodynia did not induce allodynia in recipients, thus excluding adaptive immune mechanisms (Fig. S1). We previously reported that Δ*waaL* is rapidly cleared from the bladder after an initial infection [16], and the *waaL* mutant was undetectable at 14 days following a third infection, whereas the majority of NU14-infected mice retained detectable bladder colonization despite resolution of pain (Fig. 1B). Residual NU14 colonization is consistent with previous reports of stable intracellular bacterial populations of UPEC in murine UTI [18], [19], but the absence of detectable Δ*waaL* suggests that chronic allodynia is not due to residual bacteria. Together, these findings suggest that O-antigen modulates UTI pain induced by UPEC infection, and infection has the potential to induce chronic pain.

### O-antigen modulates chronic allodynia

To exclude the influence of UPEC virulence factors in UTI pain, we examined allodynia in response to infection with the K-12 *E. coli* strain SΦ874 that bears a deletion of the entire wz* gene cluster encoding O-antigen biosynthesis genes and lacks UPEC virulence factors [20], [21]. SΦ874 caused chronic allodynia that persisted at least 35 days following a rapidly-cleared, single infection (Figs. 1C and D). Complementing the O-antigen defect in SΦ874 with the plasmid pWQ288 encoding *Klebsiella pneumoniae* O2a antigen [22] abrogated allodynia (Fig. 1C, P<0.05 Days 4–35). Despite the disparate pain phenotypes, clearance was similarly rapid for SΦ874 with/without O-antigen (45.5±43.8 CFU/bladder for SΦ874 and 55.2±36.9 CFU/bladder for SΦ874 pWQ288 at 35 days after infection, P>0.05). Moreover, four of ten SΦ874-infected mice had no detectable bladder colonization at the conclusion of the experiment while exhibiting allodynia of 459%±299 of baseline, so pain may persist even after complete bladder sterilization. These findings suggest that transient *E. coli* infection can cause chronic pain and that O-antigen modulates *E. coli* pain phenotypes in pathogens and non-pathogens alike.

**Figure 2:**
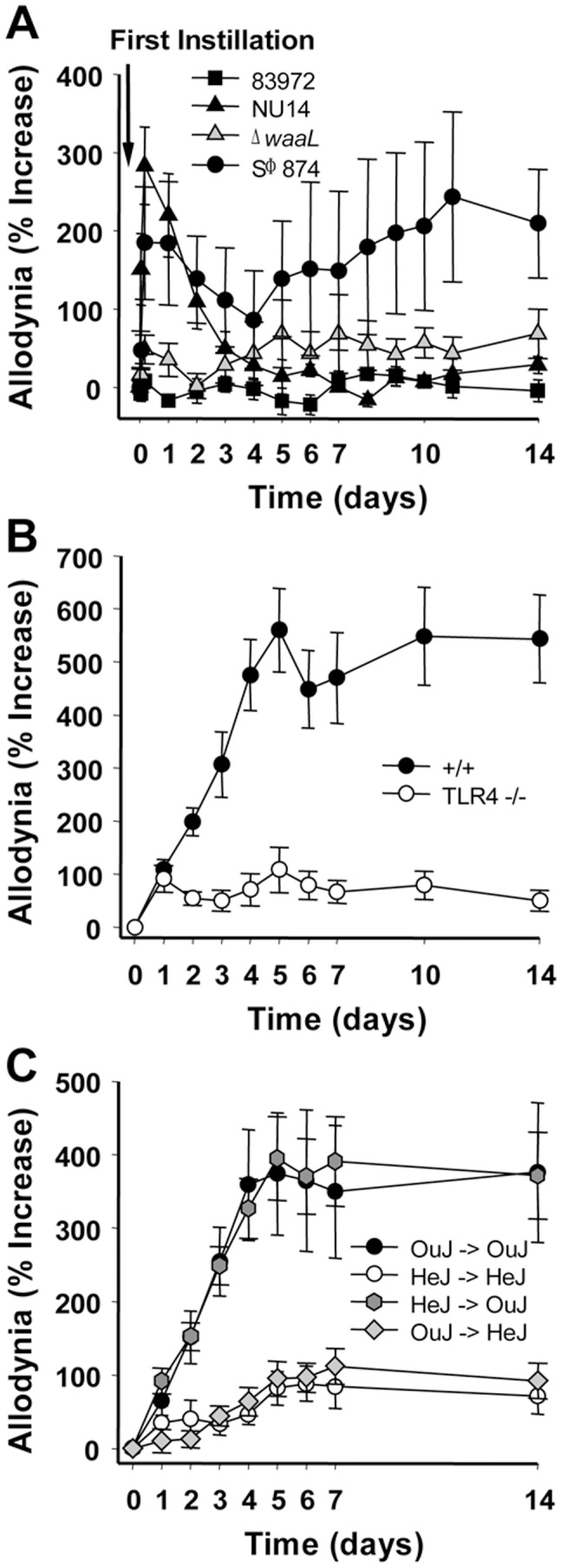
Purified LPS mimics effects of intact *E. coli*, and chronic pain is TLR4-dependent. (**A**) Mice (n = 8) were instilled 25 µl of 2 µg/ml of LPS purified from NU14, 83972, Δ*waaL*, or SΦ874 and then evaluated for pelvic allodynia. (**B**) SΦ874-induced pain in +/+ mice is reduced in TLR4^−/−^ mice (n = 5; P<0.05 Days 4–14). (**C**) +/+ mice (C3H/HeJOuJ, “OuJ") or TLR4-deficient mice (C3H/HeJ, “HeJ") were used as bone marrow donors for γ-irradiated recipients; legend arrow indicates donor bone marrow into recipient (n = 9, 9, 14 and 15 respectively). C3H/HeJOuJ recipients exhibited SΦ874-induced pain that was reduced in C3H/HeJ recipients (P<0.01 Days 3–14).

To confirm that LPS underlies the differential pain phenotypes of *E. coli*, we examined responses to bladder instillation of purified LPS. As previously reported [17], LPS from NU14 and ASB *E. coli* strain 83972 caused transient allodynia and no allodynia, respectively (Fig. 2A). In contrast, a single instillation of SΦ874 LPS induced allodynia that persisted significantly above baseline throughout the 14-day time course (Fig. 2A, P<0.05). Similar to infection with intact bacteria, purified Δ*waaL* LPS did not induce allodynia following a single instillation. Together, these data support a role for O-antigen modulation of pain and demonstrate that such modulation is not restricted to the O18 serotype of NU14 or exclusive to *E. coli* serotypes.

### Chronic allodynia is TLR4-dependent

We next examined whether chronic allodynia is TLR4 dependent, similar to TLR4-dependent acute allodynia as we previously reported for NU14 [17]. SΦ874-induced allodynia was attenuated 85.5% in TLR4-deficient C57BL/6J mice relative to +/+ mice (Fig. 2B, P<0.05 Days 4–14). The allodynia induced by Δ*waaL* serial infections was similarly attenuated in TLR4-deficient mice (65.7%; Fig. S2, P<0.05). To further demonstrate a requirement for TLR4 in chronic allodynia, we generated TLR4 chimeras by reciprocal bone marrow transplant between wild type C3H/HeJOuJ and TLR4-deficient C3H/HeJ mice. SΦ874 infection caused chronic allodynia in TLR4^+/+^ HeJOuJ recipient mice, regardless of the donor bone marrow status. In contrast, TLR4^−/−^ HeJ recipients were devoid of allodynia, even when reconstituted with TLR4^+/+^ bone marrow (Fig. 2C, P<0.05). Together, these data suggest that TLR4 mediates chronic allodynia independent of hematopoietic lineages and in multiple genetic backgrounds.

### Allodynia phenotypes are not associated with inflammation

**Figure 3:**
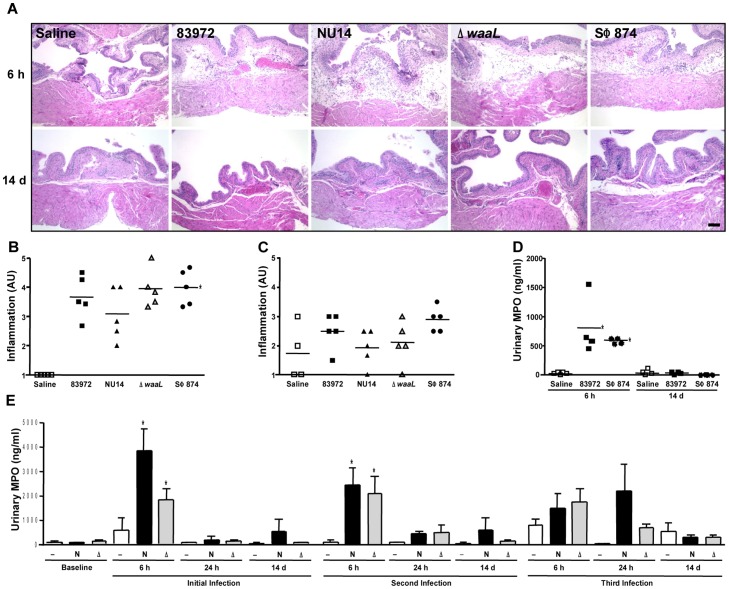
*E. coli* with differential pain phenotypes do not elicit differential inflammation. (**A**) Hematoxylin-eosin stained sections of bladders from mice instilled with saline, 83972, NU14, Δ*waaL*, and SΦ874 appeared similar at 6 hours and 14 days. Calibration mark is 100 µm. (**B**) Inflammation that was scored by a blinded reviewer and expressed as arbitrary units (AU) was significantly elevated for 83972-, NU14-, Δ*waaL-*, and SΦ874-infected bladders harvested at 6 hours, relative to saline (P<0.001) but was not significantly different among *E. coli*. (**C**) Inflammation scores were not significantly different for 83972-, NU14-, Δ*waaL-*, and SΦ874-infected bladders harvested at 14 days (P = 0.11). (**D**) Myeloperoxidase (MPO) was quantified in mouse urine by ELISA. Urines were collected at 6 h and 14 d following instillation of saline, 83972, or SΦ874. (**E**) MPO was quantified in mouse urines obtained at baseline or at 6 h, 24 h, and 14 d following serial instillation of saline (—, n = 4), NU14 (N, n = 4), or Δ*waaL* (Δ, n = 7). *P<0.05. No significant differences were observed in urinary MPO of mice with treated with NU14 or Δ*waaL.*

UPEC generate TLR4-dependent cytokine secretion and neutrophil influx [6], [23], [24], so it is possible that differential inflammatory processes underlie allodynia phenotypes modulated by O-antigen. Indeed, in a limited-inoculum model mimicking early infection, we previously observed that Δ*waaL* induces greater neutrophil influx than parental NU14 [16]. To exclude differential inflammation as the determinant of differential allodynia, we quantified bladder pathology and neutrophil influx. Scoring of tissue sections by a blinded reviewer of bladders obtained 6 hours or 14 days after instillation of 83972, NU14, Δ*waaL*, SΦ874, or saline revealed similarly elevated inflammation scores for all *E. coli* relative to saline at 6 hours after instillation that was resolved by 14 days following instillation (Figs. 3A–C, P<0.05).

Since the magnitude of acute allodynia induced by NU14 is independent of neutrophil influx [17], urinary neutrophil myeloperoxidase (MPO) was quantified to determine if chronic allodynia is somehow associated with differential neutrophil influx. A transient MPO increase was induced similarly in mice infected with 83972 or SΦ874 despite the markedly difference allodynia phenotypes (Fig. 3D). Serial infection with NU14 or Δ*waaL* also induced transient increases in urinary MPO (Fig. 3E), yet MPO was not significantly different between strains at any time point or between any single infection and serial infection. Thus, differential allodynia phenotypes that are modulated by O-antigen do not correlate with inflammation at the level of pathology or neutrophil influx.

### Infection-induced chronic allodynia is centralized

**Figure 4:**
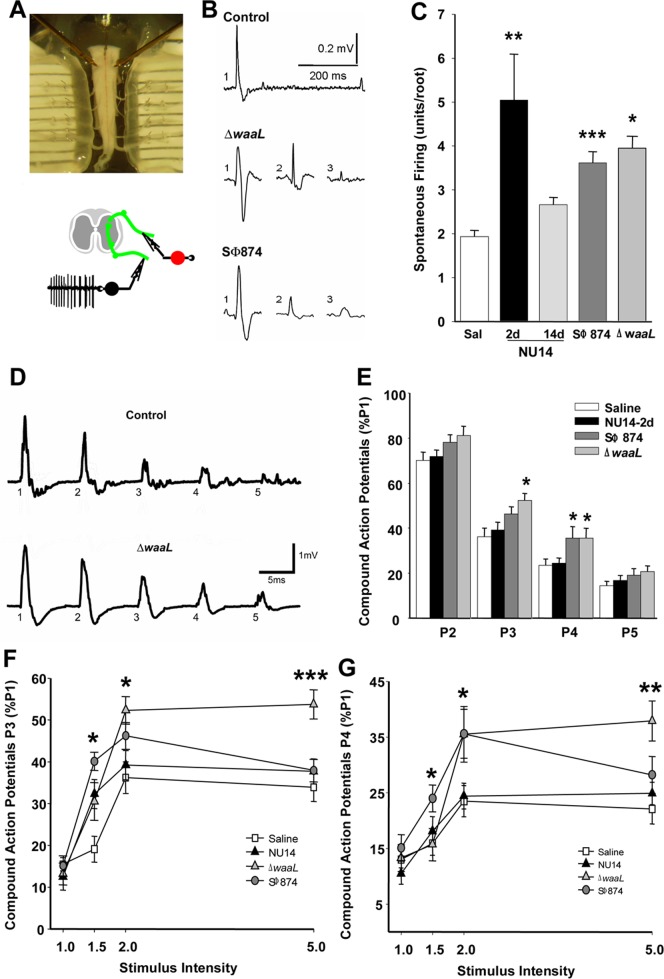
Pelvic pain behavior is associated with sacral spinal cord excitability. Spontaneous action potentials and evoked potentials were quantified in sacral spinal cords ex vivo at ventral roots S1–S3. (**A**) A sacral spinal cord is mounted in a recording chamber (upper panel), and spontaneous activity is recorded from the ventral root (lower panel). (**B**) Representative action potentials from spontaneous firing of individual neurons identified by pCLAMP. (**C**) Firing activity in sacral spinal cords is higher in Δ*waaL* at 14 d (n = 5, P = 0.0099), SΦ874 at 20 days (n = 9, P = 0.0001), and NU14 mice at 2 d (n = 6, P = 0.0298) than in saline controls (n = 7) or resolved NU14 (n = 5). (**D**) Evoked ventral root responses to dorsal root current at 2× current intensity for spinal cord of saline mouse at 2 d (upper trace) and Δ*waaL*-infected mouse after serial infection. (**E**) Normalized responses at P2–P5 relative to P1 in ventral roots of mice instilled with saline (n = 23), NU14 (n = 26), SΦ874 (n = 21), or Δ*waaL* (n = 12). *P<0.05 and **P<0.01 by Student *t* test relative to saline. (**F** and **G**) Responses across stimulus intensities at P3 and P4, respectively.

Chronic pain can result from a continuous peripheral stimulus or can be neuropathic as a result of enhanced sensory processing within the central nervous system [1]. Since SΦ874 and Δ*waaL* induce chronic allodynia persisting after bacterial clearance, we hypothesized that *E. coli*-induced chronic allodynia is associated with persistant centralizedchanges in excitability. Using an isolated murine preparation of the sacral spinal cord, the primary location of bladder sensory and micturition reflex circuits [25], [26], [27], spontaneous firing was quantified in ventral roots as a measure of coupling sensory and motor functions (Fig. 4A). This approach quantifies motor output of the flexion (withdrawal) reflex, a reflex that has long been used as a pain index [9], [28].

Spontaneous sacral activity was quantified using pCLAMP software to identify discrete action potential characteristics and thereby determine the number of spontaneously firing neurons within the flexion reflex of a spinal cord preparation (Fig. 4B). NU14 acute allodynia was associated with significantly higher spontaneous activity relative to saline controls, and this activity returned to baseline in mice with resolved allodynia at 14 days after infection, suggesting an absence of central effects (Fig. 4C, P<0.05). Spinal cords of mice with chronic allodynia from a SΦ874 or Δ*waaL* infection also exhibited significantly higher spontaneous firing relative to saline controls (P<0.001, P<0.05, respectively). These data indicate that *E. coli*-induced sacral spinal hyper-excitability is associated with allodynia and suggest that chronic allodynia has a central component.

To further examine spinal hyper-excitability, we quantified short-term depression (STD) of motor output, a measure of normal plasticity [29], by stimulating dorsal inputs and recording ventral outputs (Fig. 4A). Individual dorsal roots were stimulated with five consecutive current pulses (P1–P5) at 40 millisecond intervals at multiple stimulus intensities (Fig. 4D; 1- to 5-fold of the minimum current intensity evoking a response). For spinal cords of control mice, sequential stimuli evoked diminishing responses relative to the first pulse (P1), consistent with STD of dorsal horn sensory inputs (Fig. 4D). Responses to pulses P2–P5 were then normalized to P1 to facilitate comparisons between groups. Spinal cords from mice with acute allodynia induced by NU14 infection did not exhibit deficits in STD for any pulse or at any gain (Fig. 4E). In contrast, significant STD deficits were observed in spinal cords of mice infected with SΦ874 and Δ*waaL* that exhibited chronic allodynia (Figs. 4E–G). Both SΦ874 and Δ*waaL* spinal cords exhibited significant STD deficits at 2× stimulus intensity and at P3 and/or P4 (Fig. 4E). These STD deficits at P3 and P4 were also manifested at multiple stimulus intensities (Figs. 4F and G). These findings suggest that chronic allodynia induced by transient *E. coli* infection is associated with persistant centralized hyper-excitability characteristic of chronic pain.

### UPEC elicits diverse tactile allodynia responses via O-antigen

**Figure 5:**
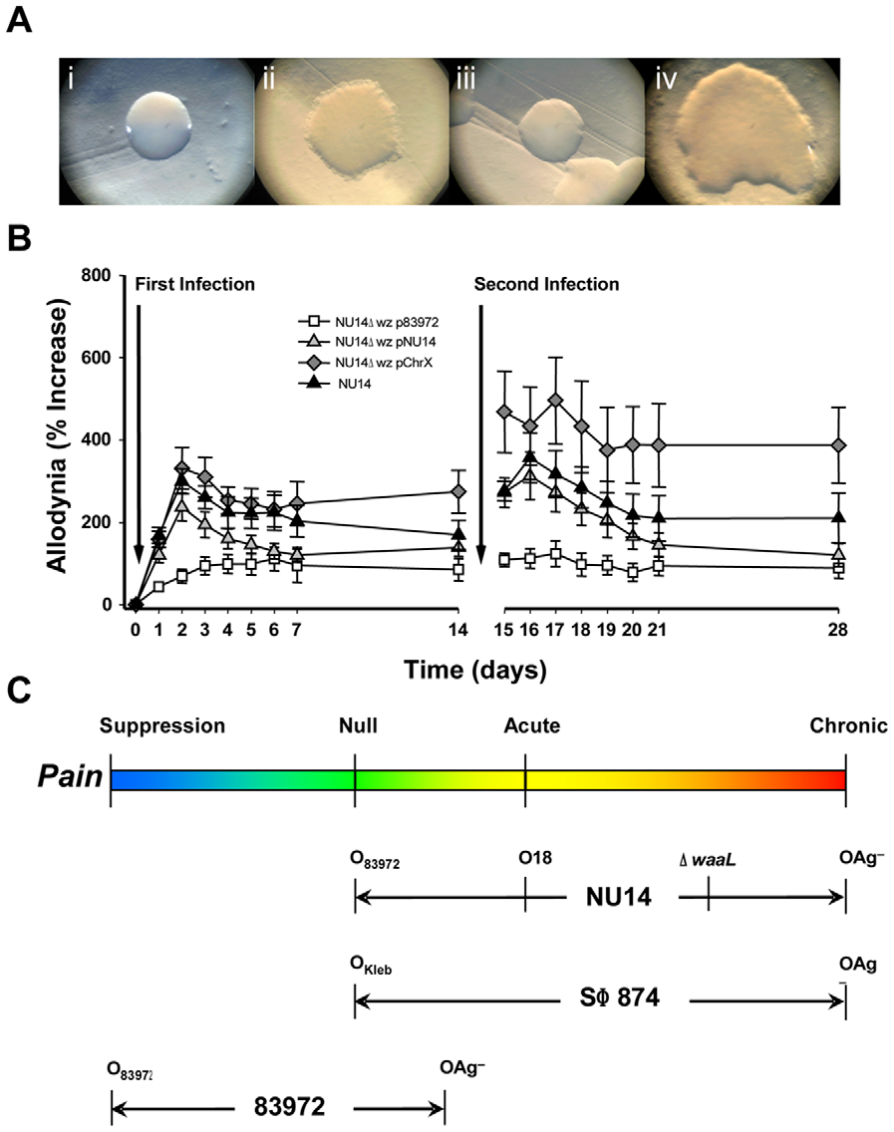
O-antigen modulates pain states. Mice were infected with NU14Δwz* bearing a deletion of the O-antigen gene clusters and harboring like or heterologous complementation constructs. (**A**) NU14 smooth colony morphology (i) is rough in the NU14Δwz* mutant with a human X chromosome plasmid (ii) or a 83972 wz* plasmid (iv) but is rescued by an NU14wz* plasmid (iii). (**B**) Tactile allodynia of mice in response to sequential infection with NU14 or NU14Δwz* containing plasmids with the wz* cluster of 83972, NU14, or a fragment of the human X chromosome (n = 10). (**C**) Summary of O-antigen modulation of pain responses.

To further characterize O-antigen modulation of pain, we deleted the NU14 wz* O-antigen gene cluster [30] to generate NU14Δ*wz*. For genetic complementation, low-copy plasmids containing the wild type wz* clusters of NU14 and 83972 were isolated from genomic libraries. The morphology of gram-negative bacterial colonies on agar is classically described as smooth in appearance for strains expressing O-antigen, whereas strains lacking O-antigen or expressing minimally-glycosylated O-antigen appear as colonies with a rough morphology (Fig. 5A). Wild type NU14 exhibited smooth morphology, whereas NU14Δ*wz* appeared rough. Complementation with the NU14 wz* gene cluster plasmid rescued the smooth phenotype. Although the 83972 wz* plasmid did not rescue the NU14 wz* phenotype, this is consistent with the absence of a typeable serotype for 83972 and a rough phenotype [31].

Serial infection with wild type NU14 or with NU14Δ*wz* complemented with the NU14 wz* plasmid elicited only acute allodynia (Fig. 5B). In contrast, NU14Δ*wz* bearing a control plasmid that contains a region of the human X chromosome induced chronic allodynia that was significantly elevated at day 14 following infection (P<0.05). Expression of the 83972 wz* cluster in NU14Δ*wz* entirely abrogated allodynia. These findings suggest that the pain phenotype of a strain can be modulated across a spectrum by O-antigen.

## Discussion

The role of O-antigen in host responses is understudied, and a role for O-antigen in modulating pain was unknown. These data demonstrate that allodynia elicited by murine UTI corresponds with spinal cord hyperexcitability, and this UTI pain response is modulated by O-antigen expression. While limited studies suggest that O-antigen modulates inflammatory responses [32], and UTI pain is TLR4-dependent, this pain appears separable from inflammation because UTI pain is independent of bladder pathology, neutrophil influx, or TLR4 expression on hematopoietic lineages ([17] and this study). TLR4-induced hypersensitivity appears specific for a given strain, since a single Δ*waaL* infection did not render mice more sensitive to subsequent instillation of NU14, 83972, or capsaicin, a known activator of bladder sensory afferents (Table S3). Conversely, a pilot experiment examining allodynia in response to an initial NU14 infection did not reveal any influence by subsequent Δ*waaL* infection relative to saline (not shown), further suggesting the specificity of inducing chronic pain. Subtle differences in initial inflammatory responses may be a co-factor contributing to differential sensory responses, since cytokines can modulate neural responsiveness [33]. However, inflammation was not different among strains with different pain phenotypes (Fig. 3). Therefore, TLR4 mediates UTI pain, and pain intensity and duration are determined *a priori* by initial TLR engagement that is subject to modulation by O-antigen.

Together, our data suggest a model whereby O-antigen acts as a rheostat that modulates LPS-associated pain across a spectrum (Fig. 5C). At its most benign with ASB strain 83972, this continuum spans from pain suppression [17] to a mild transient pain induced only by serial infection with 83972Δwz* (Fig. S3). In contrast, NU14 can exhibit a range of pain phenotypes depending upon O-antigen status: a null phenotype, acute pain, an “adaptive chronic" pain elicited by serial infection, or chronic pain following a single infection of NU14Δwz*. SΦ874 is similarly capable of either null or chronic pain phenotypes. These strain-specific differences in pain phenotypes are subject to modulation by O-antigen but are defined by other genetic factors. The LPS core oligosaccharide is one potential factor, but an *in silico* analysis based on PCR primers that discriminate among the five *E. coli* LPS core oligosaccharides [34] identified R1 as the LPS core of 83972 as (Table S4). R1 is common among UPEC and is expressed by NU14 [35], so R1 is associated with strains with pain phenotypes at either end of the spectrum. Nor is the chronic pain phenotype unique to R1 because SΦ874 expresses the K-12 core. Nonetheless, differences in O-antigen modulation of pain may mediate health and disease because gram-negative bacteria typically produce a distribution of LPS species varying in the extent of glycosylation [36], [37]. So it is possible that bacteria modulate pain phenotype during pathogenesis and/or commensal association.

The mechanism by which *E. coli* induce TLR4-dependent chronic pain may differ from other pain models. Initial infection with Δ*waaL* presumably induces hypersensitivity to a subsequent stimulus, perhaps by known mechanisms that upregulate sensory responses [38], whereas SΦ874 may induce such events in a single infection. This is reminiscent of studies where instillation of yeast zymosan into neonatal rats induces adult hypersensitivity and increased bladder content of pain-related neuropeptides [39], [40]. This is also consistent with recent findings by Farmer and colleagues where serial *Candida* infection resulted in increased peptidergic nociceptor and sympathetic fiber density in vulvar tissue, demonstrating a mechanism for peripheral hypersensitivity to infection [41]. However, that same peripheral mechanism is unlikely to mediate chronic pain in our UTI model because we did not observe increased sensory nerve fiber density in bladders of mice with chronic allodynia (data not shown).

Many microbial species coexist with human hosts without inducing pain, while different strains of the same species cause symptomatic responses for pathogens as diverse as *E. coli* and *Candida albicans*. Clinical data suggest prior infection as an etiology or sensitizing factor for chronic pain syndromes. For example, interstitial cystitis/painful bladder syndrome and vulvodynia are associated with a history of UTI and candidiasis, respectively [42], [43]. Our data thus contribute to an emerging picture of an infectious basis for at least a subset of individuals afflicted with chronic pain. We also observed TLR-dependent allodynia induced by gram-positive *S. milleri* oral bacteria (Rudick et al, in preparation). Similar to *E. coli*, *S. milleri* strains also exhibited a spectrum of pain phenotypes, from null to acute to chronic allodynia, indicating that TLR-dependent pain is a general phenomenon that extending beyond *E. coli*. Therefore, we suggest microbes evolved surface features that modulate host behaviors to their advantage through differential activation of pattern recognition receptors. Such modulation is likely widespread and extends beyond pain to other neurally-regulated processes. Indeed, *T. gondii* relieves rodents of their innate fear of cat predation by specifically altering fear of feline odors, and this is correlated with parasite cysts in the amygdala [44]. But while *T. gondii* also has been correlated with altered human behaviors, the mechanism of *T. gondii* behavioral modulation is unknown [44], [45], [46], [47]. Since *T. gondii* stimulates TLR9 [48], [49], we speculate that *T. gondii* may modulate behavior by a TLR-mediated process analogous to O-antigen-mediated pain modulation. However, in contrast to behavioral change with *T. gondii* cysts, data presented here also indicate that durable effects may persist after microbial clearance.

In summary, O-antigen modulates *E. coli* pain phenotypes to alter pain duration and severity during infection. TLR4-dependent pain may persist long after bacterial clearance, is independent of inflammation, and can result in chronic pain — supporting the possibility of an infectious etiology for chronic pain.

## Materials and Methods

### Ethics statement

All animals (mice) were housed in Northwestern's Center for Comparative Medicine and were cared for only by trained facility personnel. Mice were maintained in enriched enviroments and monitored frequently to confirm good health and absence of visible signs of stress. All experimental protocols were approved by the Northwestern IACUC and follow NIH guidelines to minimize any stress or pain, and all scientific personnel were appropriately trained and certified, including euthanasia training. All experiments were designed to minimize the number of mice needed to obtain statistically significant findings, and experiments were terminated as soon as possible.

### Mice and bacteria

Female mice were purchased from JAX at 6–12 weeks of age and maintained in the Center for Comparative Medicine and used in Northwestern IACUC-approved protocols. Bone marrow chimeras were generated by gamma irradiating recipient mice with 1000 rad total from a Cs-137 source and reconstituting with 10^7^ bone marrow cells from age-matched donors; recovery after 12 weeks of reconstitution was confirmed by LPS induction of CD80 and CD86 on splenic macrophages.

NU14 is a cystitis strain of O18 serotype, 83972 is an asymptomatic *E. coli* strain of a non-typeable serotype, and SΦ874 is a K-12 *E. coli* strain [21]. Targeted wz* deletion mutants of NU14 and 83972 were constructed using λ-Red mutagenesis with strain-specific primers spanning the *gnd* and JUMP-start sequences [50], [51]. Complementation plasmids were isolated from fosmid libraries of NU14 and 83972 in pCC1FOS (EPICENTRE Biotechnologies). A plasmid containing a 43 kb region of the human X chromosome was also isolated using the kit positive control DNA. Mutants and fosmids were confirmed by DNA sequence analysis.

### Murine UTI and pelvic pain behavior

Bacteria were cultured under static conditions and used to infect mice by instillating 10 µl containing 10^8^ CFU in a non-reflux UTI model [52]. Allodynia was quantified in response to von Frey filament stimulation to the pelvic region or the hind paw by a blinded tester [17]. Briefly, mice were adapted to the test chamber environment (5–10 min.) and von Frey filaments were applied 10 times each to the pelvic region, moving the stimulus with each successive fiber application to avoid wind up. For paw sensitivity, filaments were applied to the hind paw in a modified “up-down" stimulus protocol to establish a 50% threshold.

### Pathology scoring and MPO assay

Mice were instilled with saline or infected with 10^8^ CFU *E. coli*. Bladder sections (10 µm) were stained with hematoxylin-eosin and evaluated by a blinded reviewer. Sections were scored on a scale of 0–5 for edema, leukocytic influx, and disruptions in urothelial integrity. Data points reflect the mean scores of individual mice from an evaluation of 3 non-serial sections. MPO was quantified in urine by ELISA (Hycult Biotech).

### Sacral spinal cord recording

The spinal cord was prepared as previously described [26], [27]. Briefly, the spine was opened at upper lumbar under deep anesthesia, perfused with artificial cerebro-spinal fluid (ACSF), and then transected at the middle of the lumbar enlargement immediately after sacrifice, and the distal part of the cord with dorsal/ventral roots was transferred to a dish containing ACSF. After separating sacral ventral roots 1–3 (S1–S3) and dorsal roots on each side of the cord and removing all other roots, six recording electrodes mounted on the ventral roots (S1–S3 on each side) were connected to six DAM 50 amplifiers (WPI) in differential mode with 1000× gain, high-pass filtering at 300 Hz, and low-pass filtering at 20 kHz. Amplifier outputs were transferred to an A-to-D interface (DT1322A, Molecular Devices), and signals were digitized at 50 kHz and acquired using pCLAMP v9.1 software (Molecular Devices). Spontaneous action potentials were recorded for 30 min. For each animal, the firing units per root were the average from all recorded roots.

To record evoked motor outputs in response to dorsal stimulation, the stimulation threshold was first determined by adjusting the intensity of a 0.2 ms current pulse. Then 5 pulses at 25 Hz with intensities of 1, 1.5, 2, and 5 times the threshold stimulus were used. The evoked peak-to-peak compound action potential (coAP) was averaged for each pulse, P2–P5, relative to the coAP at P1.

### Statistical approaches

Results were expressed as means ± standard errors of the mean. Data were analyzed with the Student *t* test or ANOVA followed by a Kruskal-Wallis test, by the Dunn post test, or with analysis of variance, followed by Dunnett's post test; Prism software, version 5 (GraphPad), was used, as appropriate. Differences were considered statistically significant at P<0.05.

## Supporting Information

Figure S1 **Adaptive immune responses do not underlie Δ** ***waaL*** **-induced chronic allodynia.** (**A**) Donor +/+ were mice serially infected with Δ*waaL* exhibited chronic allodynia (n = 5). (**B–D**) CD90+ splenocytes from donor mice in (A) or saline-treated control donors were transferred to naïve recipients (n = 4 saline, n = 6 Δ*waaL*), and allodynia was quantified for four days after transfer. Pelvic sensitivity (**D**) and paw sensitivity (**E**) were unaltered by transfer of CD90 splenocytes. (**F**) Chronic allodynia following a second infection with Δ*waaL* was similar in +/+ (n = 9) and Rag1^−/−^ mice (n = 9). (**G**) At 14 days following a third infection with Δ*waaL*, bladders were harvested from mice, homogenized, and plated onto selective agar to quantify colonization. Except for a single Rag1^−/−^ mouse, no colonization was detected.
(TIF)

Figure S2 **Chronic allodynia from Δ** ***waaL*** **infection requires TLR4.** Mice (n = 10) were infected serially with Δ*waaL*, and tactile allodynia was quantified in +/+ or TLR4^−/−^ B6 mice. Allodynia following second and third infections was significantly reduced in TLR4^−/−^ mice relative to +/+ mice (P<0.01).
(TIF)

Figure S3 **O-antigen modulation of 83972-associated pelvic responses.** Tactile allodynia of mice infected with 83972 or 83972Δ*wz* with/without a plasmid encoding the wz* cluster of *K. pneumoniae* in pWQ288 in response to two sequential infections (n = 10). 83972Δ*wz* induced significant allodynia in response to serial infection that was not observed in mice receiving serial infection with 83972Δ*wz*/pWQ288 (P<0.05).
(TIF)

Table S1 Paw sensitivity determined by 50% threshold (*p<0.05). Tactile allodynia of the hind paw was assessed to determine the specificity of pelvic responses. No significant differences were detected.
(DOC)

Table S2 Body mass during infection (*p<0.05). Body mass was assessed as a marker of overall health. No significant changes were detected.
(DOC)

Table S3 **ΔwaaL-induced chronic pelvic pain (% increase).** *p<0.05 compared to all other groups at PID14. After sensitizing infection with Δ*waaL*, only Δ*waaL* resulted in chronic allodynia persisting to PID 14. Δ*waaL* did not sensitize mice to chronic allodynia from other stimuli and did not alter responsiveness to capsaicin.
(DOC)

Table S4 **In silico identification of 83972 LPS core. BLAST** analyses with conserved primer sequences known to identify E. coli sequences identified homologous sequences representing the R1 core. The region “amplified" in silico was 547 bp and corresponded to a known R1 core enzymatic function, gylcosyl transferase.
(DOC)
