# Supplementary figures and images for: O-Antigen Modulates Infection-Induced Pain States
Source: PLoS One. 2012 Aug 10;7(8):e41273. doi: 10.1371/journal.pone.0041273 (PMC3416823; doi:10.1371/journal.pone.0041273)

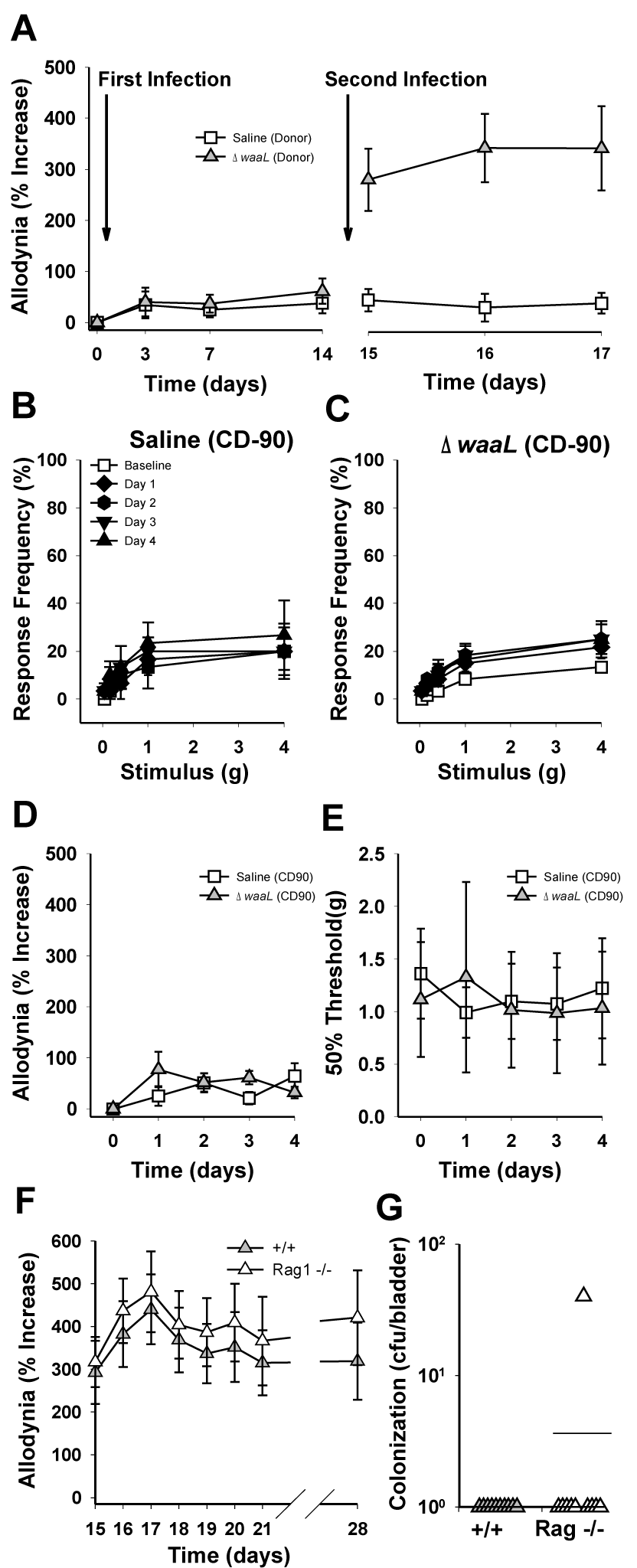

Supplement: Figure S1 — Adaptive immune responses do not underlie Δ waaL -induced chronic allodynia. (A) Donor +/+ were mice serially infected with ΔwaaL exhibited chronic allodynia (n = 5). (B–D) CD90+ splenocytes from donor mice in (A) or saline-treated control donors were transferred to naïve recipients (n = 4 saline, n = 6 ΔwaaL), and allodynia was quantified for four days after transfer. Pelvic sensitivity (D) and paw sensitivity (E) were unaltered by transfer of CD90 splenocytes. (F) Chronic allodynia following a second infection with ΔwaaL was similar in +/+ (n = 9) and Rag1−/− mice (n = 9). (G) At 14 days following a third infection with ΔwaaL, bladders were harvested from mice, homogenized, and plated onto selective agar to quantify colonization. Except for a single Rag1−/− mouse, no colonization was detected. (TIF) [file pone.0041273.s001.tif]

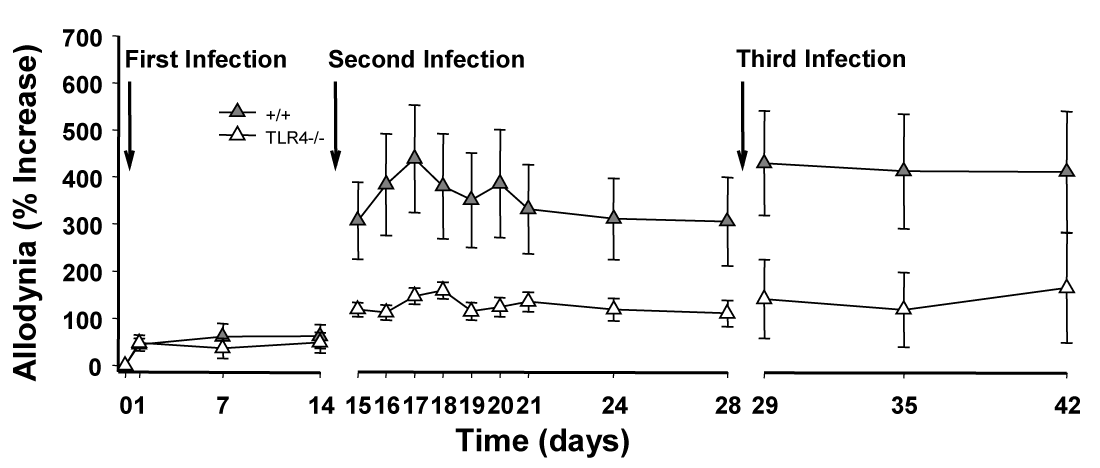

Supplement: Figure S2 — Chronic allodynia from Δ waaL infection requires TLR4. Mice (n = 10) were infected serially with ΔwaaL, and tactile allodynia was quantified in +/+ or TLR4−/− B6 mice. Allodynia following second and third infections was significantly reduced in TLR4−/− mice relative to +/+ mice (P<0.01). (TIF) [file pone.0041273.s002.tif]

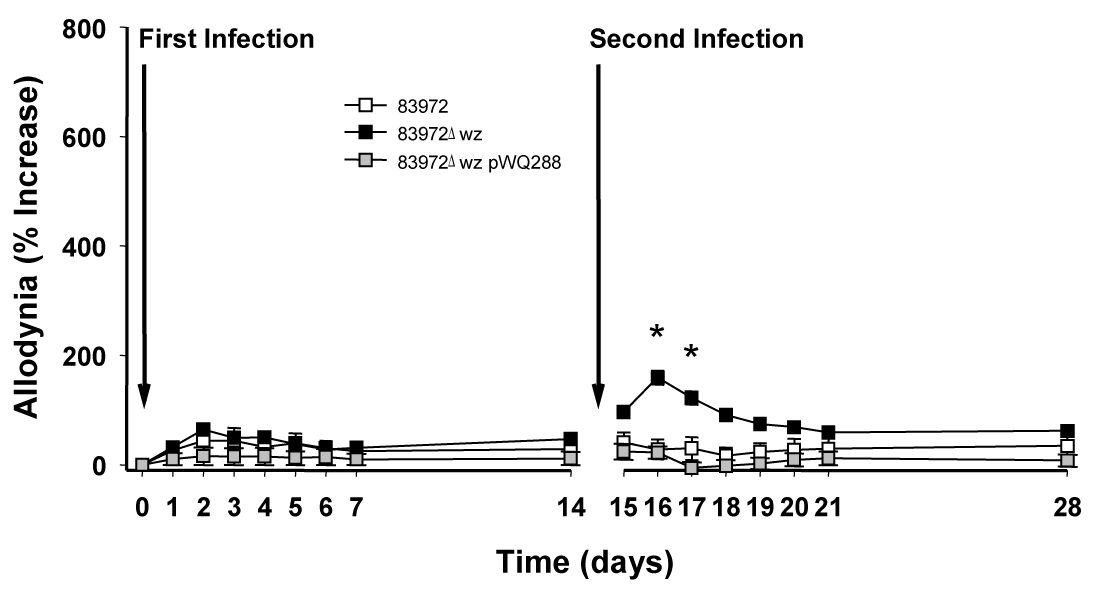

Supplement: Figure S3 — O-antigen modulation of 83972-associated pelvic responses. Tactile allodynia of mice infected with 83972 or 83972Δwz with/without a plasmid encoding the wz* cluster of K. pneumoniae in pWQ288 in response to two sequential infections (n = 10). 83972Δwz induced significant allodynia in response to serial infection that was not observed in mice receiving serial infection with 83972Δwz/pWQ288 (P<0.05). (TIF) [file pone.0041273.s003.tif]
