# Supplementary material for: O-Antigen Modulates Infection-Induced Pain States
Source: PLoS One. 2012 Aug 10;7(8):e41273. doi: 10.1371/journal.pone.0041273 (PMC3416823; doi:10.1371/journal.pone.0041273)
Supplement: Table S1 — Paw sensitivity determined by 50% threshold (*p<0.05). Tactile allodynia of the hind paw was assessed to determine the specificity of pelvic responses. No significant differences were detected. (DOC) [file pone.0041273.s004.doc]

**Table S1.** Paw sensitivity determined by 50% threshold (*p<0.05).

| Day | **Saline** | **NU14** | **Δ*waa*L** |
| --- | --- | --- | --- |
| **Baseline** | 1.07±0.32 | 1.48±0.44 | 1.08±0.24 |
| First Infection |  |  |  |
| 1 day | 1.04±0.29 | 1.12±0.23 | 1.25±0.32 |
| 2 days | 1.09±0.28 | 1.39±0.39 | 1.36±0.30 |
| 3 days | 1.42±0.52 | 1.30±0.32 | 1.27±0.32 |
| 4 days | 1.27±0.36 | 1.15±0.27 | 1.30±0.31 |
| 5 days | 1.06±0.35 | 1.16±0.27 | 1.31±0.31 |
| 6 days | 1.11±0.50 | 1.09±0.20 | 1.28±0.31 |
| 7 days | 1.16±0.32 | 1.19±0.26 | 1.20±0.26 |
| 10 days | 1.19±0.53 | 1.23±0.33 | 1.32±0.31 |
| 14 days | 1.00±0.38 | 1.14±0.19 | 1.27±0.32 |
| Second Infection |  |  |  |
| 1 day | 1.00±0.37 | 1.27±0.38 | 1.27±0.32 |
| 2 days | 1.10±0.50 | 1.02±0.22 | 1.22±0.26 |
| 3 days | 1.00±0.53 | 1.16±0.36 | 1.39±0.35 |
| 4 days | 0.94±0.35 | 1.22±0.28 | 1.20±0.26 |
| 5 days | 1.13±0.32 | 1.21±0.35 | 1.41±0.36 |
| 6 days | 1.33±0.57 | 1.14±0.29 | 1.29±0.32 |
| 7 days | 1.13±0.32 | 1.14±0.29 | 1.35±0.36 |
| 10 days | 1.10±0.35 | 1.13±0.29 | 1.44±0.35 |
| 14 days | 1.04±0.34 | 1.13±0.29 | 1.31±0.31 |
| Third Infection |  |  |  |
| 1 day | 1.22±0.58 | 1.11±0.29 | 1.39±0.36 |
| 2 days | 1.00±0.36 | 1.00±0.22 | 1.31±0.30 |
| 3 days | 1.19±0.58 | 1.10±0.30 | 1.34±0.31 |
| 4 days | 1.17±0.35 | 1.19±0.35 | 1.31±0.31 |
| 5 days | 1.04±0.34 | 0.99±0.21 | 1.38±0.36 |
| 6 days | 1.19±0.58 | 1.09±0.21 | 1.38±0.36 |
| 7 days | 1.16±0.30 | 1.28±0.36 | 1.42±0.35 |
| 10 days | 1.11±0.34 | 1.24±0.29 | 1.35±0.37 |
| 14 days | 1.10±0.34 | 1.20±0.29 | 1.18±0.26 |
