# Supplementary material for: O-Antigen Modulates Infection-Induced Pain States
Source: PLoS One. 2012 Aug 10;7(8):e41273. doi: 10.1371/journal.pone.0041273 (PMC3416823; doi:10.1371/journal.pone.0041273)
Supplement: Table S2 — Body mass during infection (*p<0.05). Body mass was assessed as a marker of overall health. No significant changes were detected. (DOC) [file pone.0041273.s005.doc]

**Table S2.** Body mass during infection (*p<0.05).

| Day | **Saline** | **NU14** | **Δ*waa*L** |
| --- | --- | --- | --- |
| **Baseline** | 18.6±0.2 | 19.5±0.4 | 18.4±0.2 |
| First Infection |  |  |  |
| 1 day | 18.9±0.2 | 19.4±0.4 | 18.5±0.2 |
| 2 days | 19.2±0.2 | 19.6±0.3 | 19.0±0.2 |
| 3 days | 19.2±0.2 | 19.7±0.4 | 19.1±0.1 |
| 4 days | 19.1±0.1 | 19.5±0.3 | 19.2±0.2 |
| 5 days | 18.8±0.2 | 19.3±0.3 | 18.9±0.2 |
| 6 days | 18.8±0.2 | 19.8±0.2 | 19.1±0.2 |
| 7 days | 18.7±0.2 | 19.6±0.3 | 18.7±0.3 |
| 10 days | 19.1±0.2 | 19.6±0.3 | 18.7±0.2 |
| 14 days | 19.1±0.1 | 19.9±0.4 | 18.6±0.2 |
| Second Infection |  |  |  |
| 1 day | 18.4±0.1 | 19.2±0.4 | 18.3±0.2 |
| 2 days | 18.8±0.2 | 19.9±0.4 | 18.8±0.3 |
| 3 days | 19.4±0.2 | 19.9±0.4 | 19.1±0.3 |
| 4 days | 19.5±0.1 | 19.8±0.3 | 19.1±0.3 |
| 5 days | 19.4±0.2 | 19.7±0.4 | 19.0±0.3 |
| 6 days | 19.4±0.3 | 20.0±0.4 | 19.1±0.2 |
| 7 days | 19.8±0.3 | 20.1±0.4 | 19.0±0.2 |
| 10 days | 19.9±0.3 | 20.2±0.4 | 19.2±0.3 |
| 14 days | 19.9±0.3 | 20.5±0.4 | 19.6±0.5 |
| Third Infection |  |  |  |
| 1 day | 20.1±0.4 | 20.2±0.4 | 19.6±0.4 |
| 2 days | 20.1±0.4 | 20.4±0.4 | 19.6±0.4 |
| 3 days | 20.1±0.4 | 20.7±0.4 | 19.8±0.3 |
| 4 days | 20.4±0.4 | 20.9±0.4 | 19.8±0.3 |
| 5 days | 19.8±0.4 | 20.6±0.4 | 19.5±0.4 |
| 6 days | 19.7±0.4 | 20.5±0.4 | 19.8±0.4 |
| 7 days | 20.6±0.3 | 20.8±0.4 | 20.0±0.4 |
| 10 days | 20.8±0.4 | 20.6±0.6 | 19.8±0.3 |
| 14 days | 19.9±0.3 | 20.6±0.4 | 20.7±0.5 |
