# Supplementary material for: O-Antigen Modulates Infection-Induced Pain States
Source: PLoS One. 2012 Aug 10;7(8):e41273. doi: 10.1371/journal.pone.0041273 (PMC3416823; doi:10.1371/journal.pone.0041273)
Supplement: Table S3 — ΔwaaL-induced chronic pelvic pain (% increase). *p<0.05 compared to all other groups at PID14. After sensitizing infection with ΔwaaL, only ΔwaaL resulted in chronic allodynia persisting to PID 14. ΔwaaL did not sensitize mice to chronic allodynia from other stimuli and did not alter responsiveness to capsaicin. (DOC) [file pone.0041273.s006.doc]

**Table S3.** ΔwaaL-induced chronic pelvic pain (% increase). *p<0.05 compared to all other groups at PID14

| Treatment | **1 hour** | **PID1** | **PID2** | **PID3** | **PID14** |
| --- | --- | --- | --- | --- | --- |
| Δ*waaL*/Δ*waaL* | - | 280 | 367 | 405 | 351* |
| Δ*waaL*/NU14 | - | 250 | 350 | 248 | 150 |
| Δ*waaL* /83972 | - | 53 | 41 | 33 | 23 |
| Δ*waaL* /capsaicin | 261 | 90 | 75 | 50 | 20 |
| Saline/capsaicin | 245 | 81 | 68 | 55 | 10 |
