# Supplementary material for: O-Antigen Modulates Infection-Induced Pain States
Source: PLoS One. 2012 Aug 10;7(8):e41273. doi: 10.1371/journal.pone.0041273 (PMC3416823; doi:10.1371/journal.pone.0041273)
Supplement: Table S4 — In silico identification of 83972 LPS core. BLAST analyses with conserved primer sequences known to identify E. coli sequences identified homologous sequences representing the R1 core. The region “amplified" in silico was 547 bp and corresponded to a known R1 core enzymatic function, gylcosyl transferase. (DOC) [file pone.0041273.s007.doc]

**Table S4.**  In silico identification of 83972 LPS core.

| **Core** | **Primer^a^** | **Sequence** | **Location^b^** | **Predicted PCR product (bp)^c^** | **PCR BLAST (identity, %)^e^** |
| --- | --- | --- | --- | --- | --- |
| R1 | R1C3 | GGGATGCGAACAGAATTAGT | 22404-22422 |  |  |
|  | R1K15 | TTCCTGGCAAGAGAGATAAG | 22935-22950 | 547 | *E. coli* H263 O-antigen polymerase glycosyl transferase 2 (100%) |
| R2 | R2C4 | GATCGACGCCGGAATTTTTT | 43296-43308 |  |  |
|  | R2K9 | AGCTCCATCATCAAGTGAGA | 36369-36380 | N.D.^d^ |  |
| R3 | R3C2 | GGCCAAAACACTATCTCTCA | 241328-241339 |  |  |
|  | R3K13 | GTGCCTAGTTTATACTTGAA | 4719-4730 | N.D. |  |
| R4 | R4C4 | TGCCATACTTTATTCATCA | 387856-387868 |  |  |
|  | R4K14 | TGGAATGATGTGGCGTTTAT | 24049-24069 | N.D. |  |
| K-12 | K12-1 | TTCGCCATTTCGTGCTACTT | 103219-103231 |  |  |
|  | K12-2a | TAATGATAATTGGAATGCTGC | 76759-76771 | N.D. |  |

^a^Primers and sequences from Amor et al (2000)

^b^Location within *E. coli* 83972 contig00007, whole genome shotgun sequence

^c^Primers and sequences from Amor et al (2000)

^d^Not determined (outside predicted size range of Amor et al)

^e^Region of 83972 contig0007 spanning upstream and downstream primer matches used in BLAST of *E. coli* genomes
